# Medical students improve their self-assessed ability in managing acute situations after simulation-based training

**DOI:** 10.1186/1757-7241-21-S2-A36

**Published:** 2013-09-09

**Authors:** Louise Simonsen, Ian Henriksen, Nicolai Helligsøe Bæk, Doris Østergaard

**Affiliations:** 1 Department of Anaesthesiology, Bispebjerg Hospital, Denmark; 2 Department of Thorax Anaesthesiology, Rigshospitalet, Denmark; 3 Danish Institute of Medical Simulation. Herlev Hospital, Denmark

## Background

Earlier studies have pointed out that medical students feel unprepared in their clinical clerkships in recognizing the acute, critically ill patient and begin initial treatment. The aim of this study was to determine if a 1- day simulation-based training course could improve medical students´ self-assessed ability to manage the acute, critically ill patient.

## Methods

Medical students in their surgical or medical clinical clerkship in the Capital Region of DK were invited to participate in one day simulator-based training course. The students were divided into groups of 4-7 students. They were trained in managing acute medical scenarios such as respiratory and/or circulatory failure with special focus on using the ABCDE assessment approach. Methods used were lectures, workshops and simulation-based training followed by feedback sessions. Before and after training, the students rated their ability to 1) perform an ABCDE assessment, 2) recognize when a patient is critically ill, 3) begin treatment of respiratory failure, and 4) of circulatory failure. Finally the students evaluated their professional development during the course, their overall benefit from the course and if they would recommend simulation training as an integrated course in their clinical clerkships.

## Results

A total of 171 students initiated the course and 160 completed the pre- and post questionnaire. The proportion of students, who rated their self-assessed ability to perform a ABCDE assessment and to recognize a critically ill patient as 'good' to 'very good' improved from 27% to 71% and from 22% to 68%, respectively.

The proportion of students who rated their ability to begin initial treatment of a patient with respiratory or circulatory failure as 'good' to 'very good' improved from 12% to 56% and from 12% to 60%, respectively.

A total of 97% of the students would recommend simulation based training as an integrated part of their clinical clerkship. A total of 90% and 95%, respectively rated their professional development and their overall course benefit as 'good' or 'very good'. P-values < 0,001.

## Conclusion

A significantly improvement in medical students´ self-assessed ability in recognizing an acutely, critically ill patient and in beginning the initial treatment were seen after a 1-day simulation based training course.

